# Intra-Tumoral Heterogeneity of HER2, FGFR2, cMET and ATM in Gastric Cancer: Optimizing Personalized Healthcare through Innovative Pathological and Statistical Analysis

**DOI:** 10.1371/journal.pone.0143207

**Published:** 2015-11-20

**Authors:** Peng Ye, Meizhuo Zhang, Shuqiong Fan, Tianwei Zhang, Haihua Fu, Xinying Su, Paul R. Gavine, Qiang Liu, Xiaolu Yin

**Affiliations:** 1 Asia & Emerging Markets iMed, AstraZeneca R&D, Shanghai, China; 2 Research & Development Information, AstraZeneca R&D, Shanghai, China; 3 Department of Pathology, Ren Ji Hospital, School of Medicine, Shanghai Jiao Tong University, Shanghai, China; Instituti Ospitalieri di Cremona, ITALY

## Abstract

Current drug development efforts on gastric cancer are directed against several molecular targets driving the growth of this neoplasm. Intra-tumoral biomarker heterogeneity however, commonly observed in gastric cancer, could lead to biased selection of patients. MET, ATM, FGFR2, and HER2 were profiled on gastric cancer biopsy samples. An innovative pathological assessment was performed through scoring of individual biopsies against whole biopsies from a single patient to enable heterogeneity evaluation. Following this, false negative risks for each biomarker were estimated *in silico*. 166 gastric cancer cases with multiple biopsies from single patients were collected from Shanghai Renji Hospital. Following pre-set criteria, 56 ~ 78% cases showed low, 15 ~ 35% showed medium and 0 ~ 11% showed high heterogeneity within the biomarkers profiled. If 3 biopsies were collected from a single patient, the false negative risk for detection of the biomarkers was close to 5% (exception for FGFR2: 12.2%). When 6 biopsies were collected, the false negative risk approached 0%. Our study demonstrates the benefit of multiple biopsy sampling when considering personalized healthcare biomarker strategy, and provides an example to address the challenge of intra-tumoral biomarker heterogeneity using alternative pathological assessment and statistical methods.

## Introduction

Gastric cancer (GC) is one of the most common cancers worldwide, with around half of all cases occurring in Eastern Asia (mainly China), and is the third leading cause of cancer-related death worldwide [1]. Although the incidence is decreasing, most GC cases are diagnosed at an advanced stage and prognosis of the disease remains poor [2]. The median survival for metastatic GC is less than one year, while the overall 5-year survival rate is less than 7% [3].

Intra-tumoral heterogeneity is commonly observed in GC. In the 1980’s, de Aretxabala *et al* evaluated 222 samples from 37 GC cases and found a mixture of diploid and aneuploid samples or different aneuploid stemlines in the same case (so called DNA content heterogeneity) in 33% of primary tumors [4]. A similar study by Yonemura *et al* showed a 69% DNA content heterogeneity in 65 resected GC samples [5]. Recently, Yang *et al* evaluated GC samples from 148 patients and found a heterogeneity rate of 79.3% in human epithelial growth factor receptor 2 (HER2) protein overexpression and 44% in *HER2* gene amplification [6]. Accordingly, the high intra-tumoral heterogeneity observed in GC is likely to contribute to treatment resistance and poorer patient prognosis [7, 8], and ultimately represents a considerable unaddressed problem faced by clinicians, pathologists, and researchers.

Several molecular targets currently feature in either approved drug treatments or promising therapeutics undergoing clinical development in GC. HER2 plays important roles in the tumorigenesis of breast cancer, ovarian cancer and gastric cancer [9] and Trastuzumab, a monoclonal antibody against HER2, has been approved for the treatment of GC [10]. The mesenchymal-epithelial transition factor (MET) gene encodes a protein which is the only known receptor for hepatocyte growth factor (HGF) ligand [11]. *MET* gene amplification and protein overexpression have been shown to lead to constant activation of the MET signaling pathway which contributes to tumor growth, angiogenesis and metastasis [12]. Several MET inhibitors are currently undergoing GC clinical trials, including Savolitinib (Phase 1 (NCT02252913)[13]) and AMG337 (Phase 2 (NCT02016534)). Similarly, fibroblast growth factor receptor 2 (FGFR2) is also implicated in cell proliferation, differentiation and motility, and amplification of the *FGFR2* gene plays an important role in the tumorigenesis of GC, thereby underscoring its attraction as drug development target [14–16]. Ataxia telangiectasia mutated (ATM) is a protein kinase belonging to the phosphatidylinositol 3’ kinase (PI3K) family, and under normal conditions is activated in response to DNA double-strand breaks [17]. ATM deficiency is related to a high incidence of tissue malignancies [18–20] and ATM-deficient tumors cells are sensitive to poly (ADP-ribose) polymerase-1 (PARP) inhibition, a potential target which has been proposed for the treatment of GC in several previous studies [21–24]. Lynparza, the first US and European-approved PARP inhibitor targeting BRCA1/2 mutant ovarian cancer, is currently undergoing a Phase III clinical trial in GC (NCT01924533) and is employing a patient selection biomarker approach using ATM expression by IHC (publication in press).

In the current era of molecularly targeted drug development, biomarkers are expected to precisely predict clinical response [25]. High tumor heterogeneity however, may lead to a biomarker detection bias if the samples are obtained from a small tumor region rather than the whole tumor tissue (e.g. surgically resected samples are usually 2 cm x 2cm only). In contrast, biopsy samples are usually obtained from different regions of the whole tumor and are likely to be more representative of the patients’ overall biomarker expression status, arguing for their potential to reduce the impact of intra-tumoral heterogeneity on patient selection bias.

In our study, in order to better evaluate intra-tumoral heterogeneity, we employed surgical biopsy as our tumor sampling strategy. In addition, we performed an innovative pathological assessment through scoring of individual biopsies against whole biopsies from single patients. Herein, we also employed statistical methods to estimate the false negative detection risks when analyzing finite numbers of biopsies in order to understand the relationship between the number of biopsies and the risk of selecting a false positive patient for a particular treatment or inclusion in a clinical trial.

## Materials and Methods

### Patient information

Archived GC biopsy samples were collected from 166 patients who received gastroscopy examination with multiple biopsies from different tumor areas of each patient between 2007 and 2014 at Renji hospital, Shanghai, China. Prior written informed consent was obtained from all patients and the study protocol was approved by the Renji Hospital Institutional Review Board. All samples were reviewed by two trained pathologists for GC diagnosis and forty samples were excluded in the study due to poor tissue quality.

### Immunohistochemistry (IHC)

Formalin fixed and paraffin embedded (FFPE) samples were sectioned at 4μm thickness. For MET staining, a rabbit monoclonal anti-total MET antibody (cMET SP44, Ventana Medical Systems, AZ, USA) was used and the assay was performed on an automatic stainer (Discovery XT, Ventana Medical Systems, AZ, USA). ATM staining was performed using a rabbit monoclonal anti-ATM antibody (ab32420, Abcam, MA, USA) on an autostainer (Thermo Scientific, MA, USA). HER2 staining was performed using the HercepTest kit (DAKO, Denmark) as per the manufacturer’s instructions on an automatic stainer (Discovery XT, Ventana Medical Systems, AZ, USA).

### Fluorescence in situ hybridization (FISH)

The dual-color FISH assay was performed as previously described [26]. *HER2/CEP17* probes were purchased from Vysis (IL, USA; Cat. #30–171060). *MET* and *FGFR2* probes were prepared by labelling BAC (CTD-2270N20 and RP11-62L18, respectively) DNA with Red-dUTP (Enzo Biochem, NY, USA; Cat. #02N23-050), *CEP10*-Spectrum Green and *CEP7*-Spectrum Green probes were purchased from Vysis (Cat. # 32–112010 and # 32–132007, respectively) and used as internal controls for *FGFR2* and *MET* probes.

### Pathology assessment on biopsies

**Fig 1:**
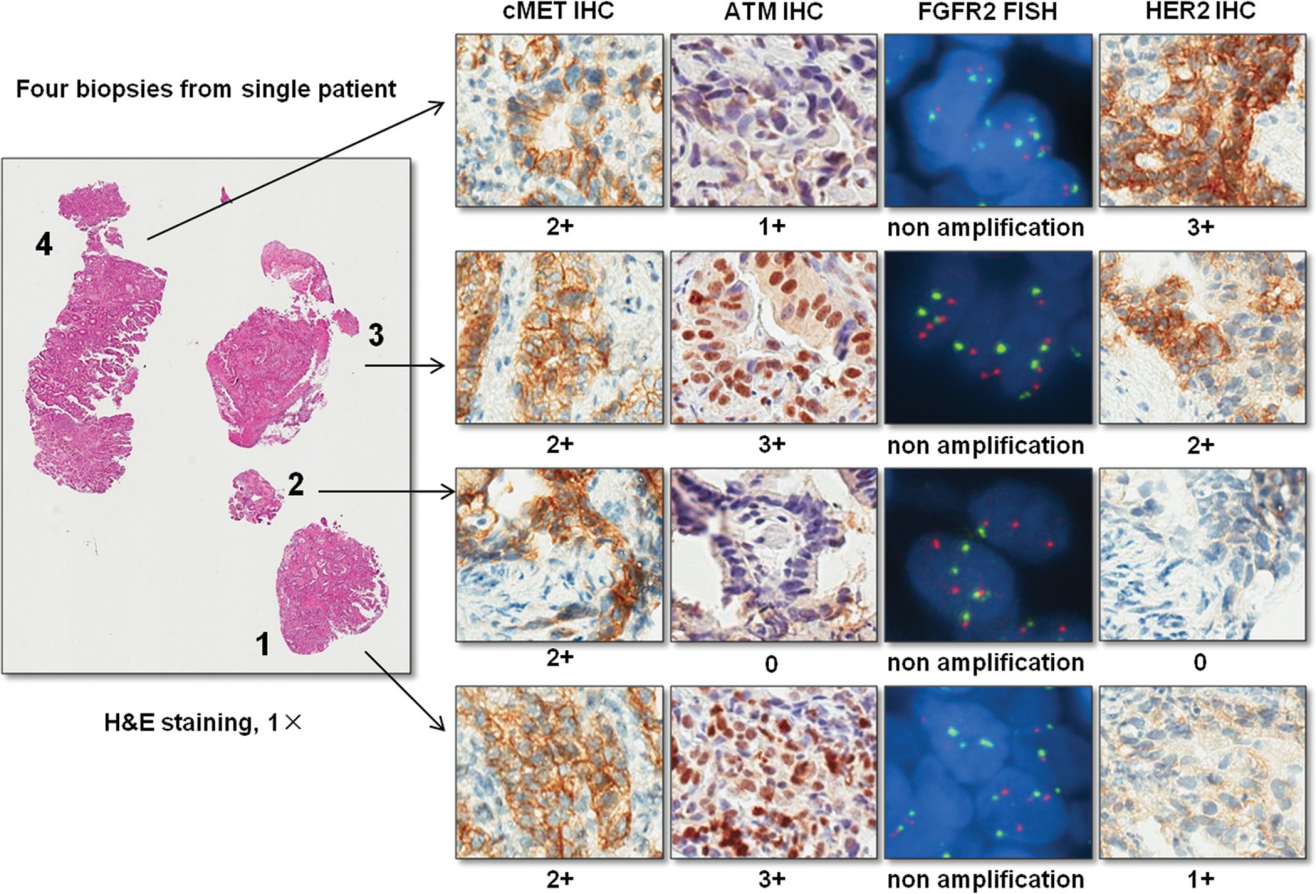
Pathological assessment of biomarker status on each individual biopsy. This is an example case which shows the individual score given to each biopsy for each biomarker. In addition, the figure also shows the heterogeneity of biomarker status between different biopsies in the same case.

Based on H&E staining, each biopsy with adequate tumor cells (more than 50 tumor cells) was firstly marked by a pathologist. Then biomarker status including IHC and FISH staining of MET, ATM, FGFR2 and HER2 were evaluated on each biopsy. Individual scores for each biomarker were given to each biopsy (Fig 1).

According to MetMab trial in GC (NCT01662869), for MET IHC staining, a biopsy showing IHC 3+ is defined as positive; for MET FISH, biopsy showing *MET* gene average copy number ≥ 5 is defined as positive. Since the ongoing trial for another MET inhibitor, AZD6094 (NCT02449551), uses *MET* gene average copy number ≥ 4 as cut off for single agent treatment arm on GC patients, we further divided the MET FISH negative group into two subgroups (*MET* gene average copy number ≥ 4 and < 5, and *MET* gene average copy number < 4). For ATM IHC staining, biopsy showing IHC 0 is defined as negative according to Olaparib trial (NCT01063517). For FGFR2 FISH, biopsy showing *FGFR2* gene amplification (average copy number ≥ 6) is defined as positive according to trials of AZD4547 (NCT01457846) and dovitinib (NCT01719549). For HER2, biopsy showing HER2 IHC 3+ or HER2 IHC 2+ plus *HER2* gene amplification is defined as positive according to ToGA trial (NCT01041404).

For MET IHC, MET FISH, FGFR2 FISH and HER2, cases with *any* of the biopsies showing positive are defined as positive cases. For ATM IHC staining, cases with all biopsies showing negative are defined as negative cases.

### Heterogeneity degree assessment

After pathologist’s review, the degree of biomarker heterogeneity was determined according to the following criteria:

High heterogeneity: < 25% of biopsies with MET IHC 3+, *MET* gene amplification, ATM IHC 0, *FGFR2* gene amplification, or HER2 positivity.

Medium heterogeneity: 25% ~ 50% of biopsies with MET IHC 3+, *MET* gene amplification, ATM IHC 0, *FGFR2* gene amplification, or HER2 positivity.

Low heterogeneity: ≥50% biopsies with MET IHC 3+, *MET* gene amplification, ATM IHC 0, *FGFR2* gene amplification, or HER2 positivity.

For MET IHC, MET FISH, FGFR2 FISH, and HER2 positivity, the mean percentages of positive biopsies in an individual case among the positive cases were calculated. For ATM IHC, the mean percentage of ATM IHC negative biopsies in an individual case amongst cases with at least one ATM negative biopsy was calculated. The 95% confidence intervals of the above mean values were assessed by bootstrapping.

### False negative detection risk assessment

For each biomarker and a predefined number of biopsies *n* (0< n < maximum number of biopsies from a sample), all possible scenarios of choosing *n* biopsies from each sample and making a determination of the biomarker’s status for the sample based on the *n* chosen biopsies were generated computationally.

Based on the scenarios enumerated above, the risks of false negative detection were assessed. For MET IHC, MET FISH, FGFR2 FISH, and HER2 positivity, the risk of false negative detection with *n* biopsies from each sample was defined as the expected number of the ratio between the number of positive samples that have negative detection results with *n* biopsies and the total number of positive samples. For ATM IHC, the false negative detection risk with *n* biopsies from each sample was defined as the expected value of the ratio between the number of non-negative samples with all negative biopsies with *n* biopsies from each sample, and the total number of ATM non-negative samples.

All computations were exact except ATM IHC with one biopsy from each sample because of the extremely large number of possible scenarios. For ATM IHC with one biopsy from each sample, the risk of false negative detection was estimated by taking a random subset of 30 non-negative samples without replacement at a time, computing the risk of false negative detection in the subset, repeating the process 22,000 times and taking an average of the risks of false negative detection from the 22,000 random subsets. In addition, the 95% Confidence Interval of this estimated risk was reported.

## Results

### Overview of GC biopsy numbers in clinical samples

**Fig 2:**
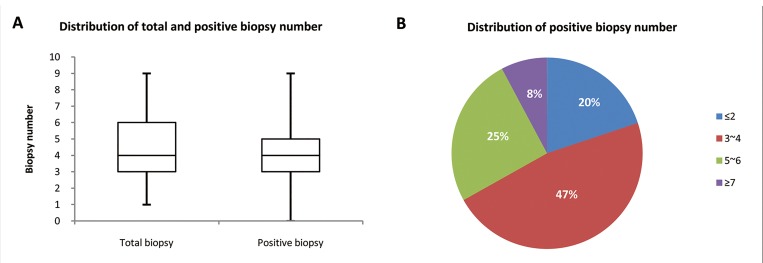
Overview of biopsy number in clinical samples. **(A) Distribution of total and positive biopsy number.** In this Chinese GC cohort, total biopsy numbers range from 1 to 9, with a median of 4. Positive biopsy (biopsy with tumor) is slightly lower than total biopsy number. **(B) Distribution of positive biopsy number.** Majority of biopsy numbers fall into 3~4 (47%) and 5~6 (25%).

In this cohort, the number of biopsy samples from a single patient ranged from 1 to 9, with the median of both total and positive biopsies (with tumor cells) at 4. The positive biopsy numbers were slightly less than the total biopsy numbers. Cases with 3~4 and 5~6 positive biopsies accounted for 47% and 25% respectively of all the samples collected (Fig 2).

### Heterogeneity degree and false negative assessment

**Fig 3:**
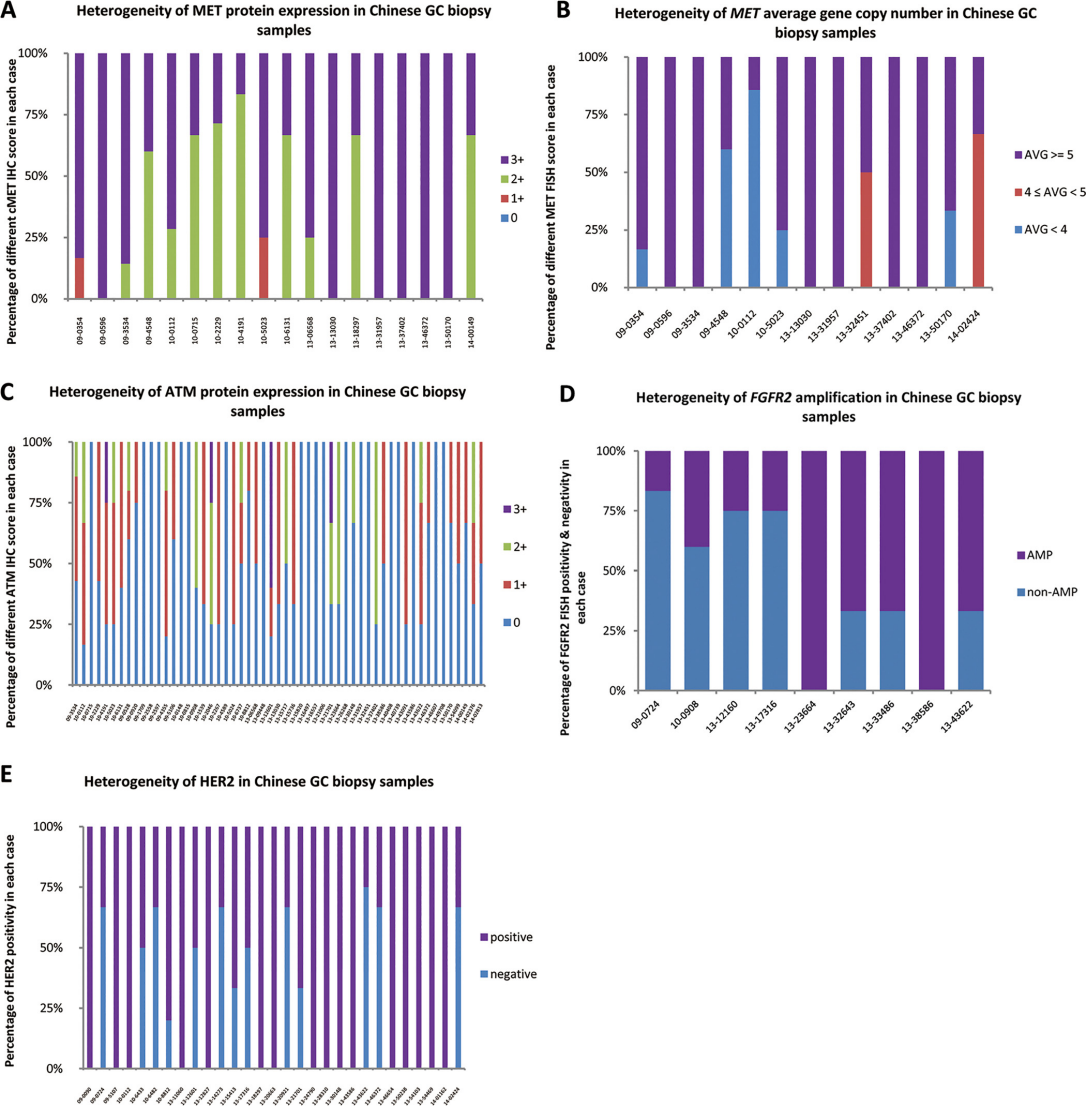
An illustration of heterogeneity distribution of each biomarker. Heterogeneity distribution of MET protein expression **(A)**, *MET* average gene copy number **(B)**, ATM protein expression **(C)**, *FGFR2* amplification **(D)**, and HER2 positivity **(E)**. AMP: amplification. AVG: average copy number.

**Fig 4:**
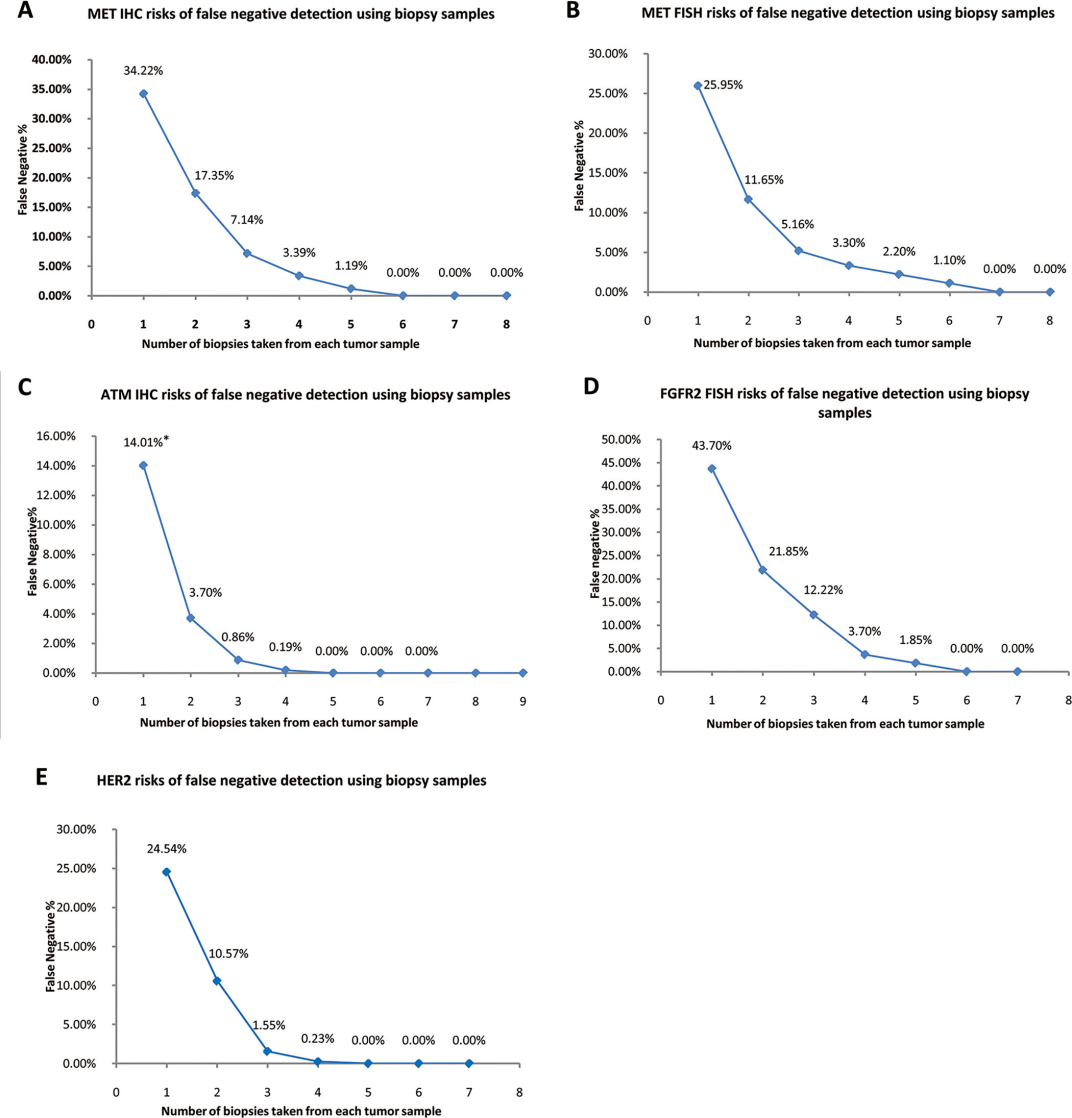
Risk assessment against different biopsy numbers in each biomarker. The risks of false negative detection along with different biopsy numbers in MET IHC **(A)**, MET FISH **(B)**, ATM IHC **(C)**, FGFR2 FISH **(D)**, and HER2 **(E)**. **Note:** *Estimated by resampling, 95% CI: 7.5%–20.94%.

In the 18 MET IHC positive cases (Fig 3A), 61% of the cases showed low heterogeneity, while 33% showed medium and 5.5% showed high heterogeneity. The mean percentage of MET positive biopsies in an individual case amongst those 18 positive cases was 65.78% (95% CI: 52.14%–79.60%). The MET false negative detection rate was estimated at around 3.39% with 4 biopsies and approached 0% when sampling 6 biopsies (Fig 4A).

In the 13 MET FISH positive cases (Fig 3B), 77% of the cases showed low heterogeneity, while 15% showed medium and 8% showed high heterogeneity. The mean percentage of MET FISH positive biopsies in an individual case amongst those 13 positive cases was 74.05% (95%CI: 57.53%–89.10%). The MET FISH false negative detection rate was estimated at around 3.30% with 4 biopsies and approached 0% when sampling 7 biopsies (Fig 4B). In addition, significant correlation was found between MET IHC score and MET FISH results (p < 0.01, κ = 0.62, Fisher’s exact test)

In the 58 cases with at least one ATM negative biopsy (Fig 3C), 62% of the cases showed low heterogeneity, while 35% showed medium and 3.6% showed high heterogeneity. The mean percentage of ATM IHC negative biopsies in an individual case amongst those 58 cases was 63.07% (95%CI: 54.93%–71.39%). The ATM IHC false negative detection rate was estimated at around 0.19% with 4 biopsies, and approached 0% with 5 biopsies (Fig 4C).

In the 9 FGFR2 FISH positive cases, 56% of the cases showed low heterogeneity, while 33% showed medium and 11% showed high heterogeneity (Fig 3D). The mean percentage of FGFR2 FISH positive biopsies in an individual case amongst those 9 positive cases was 56.30% (95%CI: 36.85%–76.85%). The FGFR2 FISH false negative detection rate was estimated at around 3.70% with 4 biopsies and approached 0% with 6 biopsies (Fig 4D).

In the 32 HER2 positive cases, 78% of the cases showed low heterogeneity, while 22% showed medium heterogeneity and none of the cases showed high heterogeneity (Fig 3E). The mean percentage of HER2 positive biopsies in an individual case amongst those 32 positive cases was 75.16% (95%CI: 65.88%–85.11%). The HER2 false negative detection rate was estimated at around 0.21% with 4 biopsies and approached 0% with 5 biopsies (Fig 4E).

## Discussion

Intra-tumoral biomarker heterogeneity has long been an issue in the selection of patients for clinical trials and therefore, understanding tumor heterogeneity is crucial to the successful deployment of a personalized healthcare biomarker (PHB) strategy. However, few studies have so far addressed this problem and there is no standardized strategy in measuring the degree of tumor heterogeneity. In this study, we took a novel approach by scoring each individual biopsy and calculating the level of heterogeneity within each case. Our results showed that high levels of heterogeneity were only found in 0 ~ 11% of the positive (or negative for ATM) cases, while most positive cases (56% ~ 78%) showed low heterogeneity, indicating a relatively low level of heterogeneity for our selected biomarkers in this cohort of GC cases.

In addition, we also performed false negative assessments for each biomarker to estimate the false negative rates associated with collecting various numbers of biopsies. Results showed that when 3 or more biopsies were collected, the false negative risks were close to 5% for all tested biomarkers (7.14%, 5.16%, 0.86%, and 1.41% respectively for MET IHC, MET FISH, ATM IHC, and HER2). This number (3–4 biopsies) is roughly equivalent to the average number of biopsies collected in clinical practice for this cohort and as such, indicates the relatively low false negative risk associated with these biomarkers in our cohort. One exception that FGFR2 FISH showed a higher false negative rate (12.2% false negative rate for 3 biopsies), could be due to the limited FGFR2-positive sample size (9 positive samples). When a total of 6 biopsies were collected from a single patient, the false negative risk for MET, ATM, FGFR2 and HER2 approached 0% in this cohort. These results provide an example of how increasing biopsy numbers could be used to address the challenge of biomarker heterogeneity in deploying clinical patient selection approaches.

Considering the importance of accurate patient selection in clinical trials, we firmly believe that adequately addressing biomarker heterogeneity is critical to success. For example, MetMab showed a significant improvement in both progression-free survival (2.9 vs. 1.5 months) and overall survival (12.6 vs. 3.8 months) [27] in a phase 2 trial (NCT01590719) however, this improvement did not successfully transfer to the phase 3 setting (NCT01662869). Notably, MET protein overexpression (by IHC) was selected as a patient selection criteria [28]. Although still an issue of debate, it is possible that the promise of MetMab in phase 2 but its failure in phase 3 was at least in part a consequence of tumor heterogeneity, and an inability of the patient selection strategy (IHC) to robustly address the challenge of intra-tumoral heterogeneity in GC.

Finally, we have also compared the positivity rate (or negativity rate for ATM) of the biomarkers detected in this cohort of biopsy samples with the surgical samples from our previous studies (Table 1). With the exception of ATM, both biopsy and surgical samples were collected from the same local hospital. Results showed that although positivity rates are higher (for ATM, negativity rates are lower) in biopsy samples, the overall results in biopsy samples were similar to surgical samples. This increase in positivity rate (or decrease in negativity rate for ATM) is likely explained by the detection of positive cases using multiple biopsies which were missed using previous sampling strategies (ie. surgical resections).

**Table 1:** Comparison of biomarker positive/negative rates using either surgical or biopsy samples. The positive/negative rate for each biomarker is comparable between the biopsy samples in this study and surgical samples profiled in our previous studies, including ATM [29] and other biomarkers [26]. A slight increase in positive rates for the biomarkers (or decrease in negative rate for ATM) was observed, which could possibly be explained by the detection of positive samples which were previously missed when analyzing surgical samples due to intra-tumoral heterogeneity. Both biopsy and surgical samples were collected from the same local hospital [26], except for ATM [29].

Biomarker positivity/negativity	Surgical sample	Biopsy sample
**MET IHC positive rate**	12.2%	14.0%
**MET FISH positive rate**	6.1%	10.3%
**FGFR2 positive rate**	5.2%	7.3%
**HER2 positive rate**	13.4%	23.0%
**ATM negative rate**	13.9–16.0%	14.7%

Taken together, this study has addressed the challenge of tumor heterogeneity from an innovative angle by using biopsies as the tumor sampling approach and giving individual biomarker scores to each biopsy. Our results show a relatively low level of heterogeneity across the biomarkers analyzed in this cohort. Nevertheless, the degree of heterogeneity within other patient cohorts may be different and should be analyzed on a case-by-case basis. Furthermore, our results showed a decrease in the rate of false negative detection corresponding with an increase in the biopsy number for all biomarkers tested herein, demonstrating the benefit of multiple biopsy sampling and serving as an example of addressing intra-tumoral heterogeneity using statistical methods.
